# Virtual Reality-Assisted Awake Craniotomy: A Retrospective Study

**DOI:** 10.3390/cancers15030949

**Published:** 2023-02-02

**Authors:** Florian Bernard, Anne Clavreul, Morgane Casanova, Jérémy Besnard, Jean-Michel Lemée, Gwénaëlle Soulard, Renaud Séguier, Philippe Menei

**Affiliations:** 1 Département de Neurochirurgie, CHU Angers, 49933 Angers, France; 2 Laboratoire d’Anatomie, Faculté de Médecine, 49045 Angers, France; 3 Université d’Angers, Inserm UMR 1307, CNRS UMR 6075, Nantes Université, CRCI2NA, 49045 Angers, France; 4 FAST/IETR Team, CentraleSupélec, 35700 Rennes, France; 5 Département de Psychologie, Université d’Angers, 49045 Angers, France

**Keywords:** virtual reality, awake craniotomy, visual field, social cognition, visuospatial cognition, unilateral spatial neglect

## Abstract

**Simple Summary:**

Awake craniotomy (AC) with brain mapping by direct electrical stimulation for tumors within or adjacent to eloquent brain regions is a surgical approach for minimizing the risk of postoperative neurologic deficits and preserving the patient’s health-related quality of life. Language and motor functions are frequently mapped, but mapping is less frequent for more complex functions, such as visuospatial and social cognition, despite the importance of these functions for daily life activities. This lack of mapping for these functions results at least in part from a lack of tasks fully compatible with the restrictive environment of an operating room and AC procedures. We show here that the use of a virtual reality headset with eye tracking opens up new possibilities for the mapping of these complex functions.

**Abstract:**

Background: Awake craniotomy (AC) with brain mapping for language and motor functions is often performed for tumors within or adjacent to eloquent brain regions. However, other important functions, such as vision and visuospatial and social cognition, are less frequently mapped, at least partly due to the difficulty of defining tasks suitable for the constrained AC environment. Objective: The aim of this retrospective study was to demonstrate, through illustrative cases, how a virtual reality headset (VRH) equipped with eye tracking can open up new possibilities for the mapping of language, the visual field and complex cognitive functions in the operating room. Methods: Virtual reality (VR) tasks performed during 69 ACs were evaluated retrospectively. Three types of VR tasks were used: VR-DO80 for language evaluation, VR-Esterman for visual field assessment and VR-TANGO for the evaluation of visuospatial and social functions. Results: Surgery was performed on the right hemisphere for 29 of the 69 ACs performed (42.0%). One AC (1.5%) was performed with all three VR tasks, 14 ACs (20.3%) were performed with two VR tasks and 54 ACs (78.3%) were performed with one VR task. The median duration of VRH use per patient was 15.5 min. None of the patients had “VR sickness”. Only transitory focal seizures of no consequence and unrelated to VRH use were observed during AC. Patients were able to perform all VR tasks. Eye tracking was functional, enabling the medical team to analyze the patients’ attention and exploration of the visual field of the VRH directly. Conclusions: This preliminary experiment shows that VR approaches can provide neurosurgeons with a way of investigating various functions, including social cognition during AC. Given the rapid advances in VR technology and the unbelievable sense of immersion provided by the most recent devices, there is a need for ongoing reflection and discussions of the ethical and methodological considerations associated with the use of these advanced technologies in AC and brain mapping procedures.

## 1. Introduction

Brain mapping by direct electrical stimulation (DES) during awake craniotomy (AC) is generally used to evaluate language and motricity in order to preserve these functions [1,2,3,4,5]. For this reason, AC is mostly performed on the left hemisphere, in which most language processing occurs. However, regardless of the side of the brain on which tumors occur, they have a similar effect on the patient’s perceived quality of life [6,7,8,9]. This probably reflects the effects on other important cognitive functions, such as visuospatial and social cognition, which are mostly right-lateralized [10,11]. Visuospatial cognition supports spatial awareness, perception and the representation of space. Lesions of the network underlying visuospatial cognition are associated with various symptoms, the most common being unilateral spatial neglect (USN). USN is typically associated with right hemisphere damage. It is defined as a failure to report, respond to, or orient in response to stimuli delivered in the space contralateral to the lesion in patients with brain damage. Social cognition encompasses all the cognitive processes involved in social interaction through non-verbal communication, such as the recognition of facial emotion, emotional prosody, eye gaze, empathy and theory of mind (TOM). Empathy is the ability to understand and feel another’s emotions and TOM is the ability to understand and act according to the mental states (beliefs, intentions and desires) of other humans [12]. Visual field defects after tumor surgery can also affect the patient’s perceived quality of life. Permanent hemianopia, which has been largely underestimated by neurosurgeons, is a significant postoperative handicap that impairs daily life activities, including, in particular, the ability to drive a motor vehicle or to read [13,14,15].

Far fewer procedures have been published for the mapping of optic radiation and visuospatial and social cognition than for language and motor mapping [14,16,17,18,19,20,21,22,23,24,25,26,27,28]. This may be explained by the complexity of the corresponding functional anatomy. Unlike the motor areas of the brain, visuospatial and social cognition networks and verbal language networks cannot be localized on the basis of anatomical criteria alone. Indeed, there are structural and functional variations within and between subjects [10,29]. The lack of tasks fully compatible with the restrictive environment of an operating room and AC procedures provides another explanation for the lack of mapping for certain functions [30]. It may be difficult to find a space directly in front of the patient and the patient’s position may not be suitable for performing the tasks. The patient must also give an unambiguous answer within five sec, the maximum duration of DES.

Virtual reality (VR) has boomed in recent years, with increasing numbers of applications for this technology emerging in the field of cognitive and social neurosciences [31,32,33,34]. This computer technology generates realistic images simulating the physical presence of the user in a virtual environment. Access to this technology has increased recently, and virtual reality headsets (VRHs) in particular are now affordable and can be installed in front of the patient’s head [13,35,36,37]. In this retrospective study, we aimed to use illustrative cases to demonstrate the potential of VR to open up new possibilities for the mapping of language, the visual field and complex cognitive functions during AC.

## 2. Methods

### 2.1. Patients

We performed a single-center, retrospective study on 64 patients undergoing AC with brain mapping through VR tasks. All the patients came from two clinical trials: ClinicalTrials.gov NCT03010943 and NCT04288505. These two trials were approved by the ANSM (“Agence nationale de sécurité du médicament et des produits de santé”), the ethics committee and the CNIL (“Commission Nationale de l’Informatique et des Libertés”) (NCT03010943: ID-RCB: 2016-A01139-42, ethics committee: CPP OUEST II, date of approval: 18 October 2016; NCT04288505: ID-RCB: 2020-A00074-35, ethics committee: CPP Est II, date of approval: 17 April 2020). All patients signed a written informed consent form.

### 2.2. VRHs

One of the difficulties in the field of VR research is the rapid technological progress in this field, leading to the regular release of new VRHs. Since we first began using VR in 2014, we have successively used a number of different types of VRHs: the Oculus VRHs DK1 and DK2 (visual field: 100°, resolution: 1280 × 800, refresh rate: 60 Hz) (Oculus, Menlo Park, CA, USA); the Samsung Gear VR combined with a Samsung S7 smartphone (Android platform) (visual field: 96°, resolution: 1440 × 1280, refresh rate: 60 Hz); and the HTC Vive (visual field: 110°, resolution: 2160 × 1200, refresh rate: 90 Hz) combined with an eye-tracking device (Tobii Pro SDK, Danderyd, Sweden). The eye-tracking system collects data at a rate of 120 Hz for various aspects of eye movement, including gaze origin and direction, pupil position and absolute pupil size, with an accuracy of 0.5° in visual angle.

### 2.3. VR Tasks

**Figure 1:**
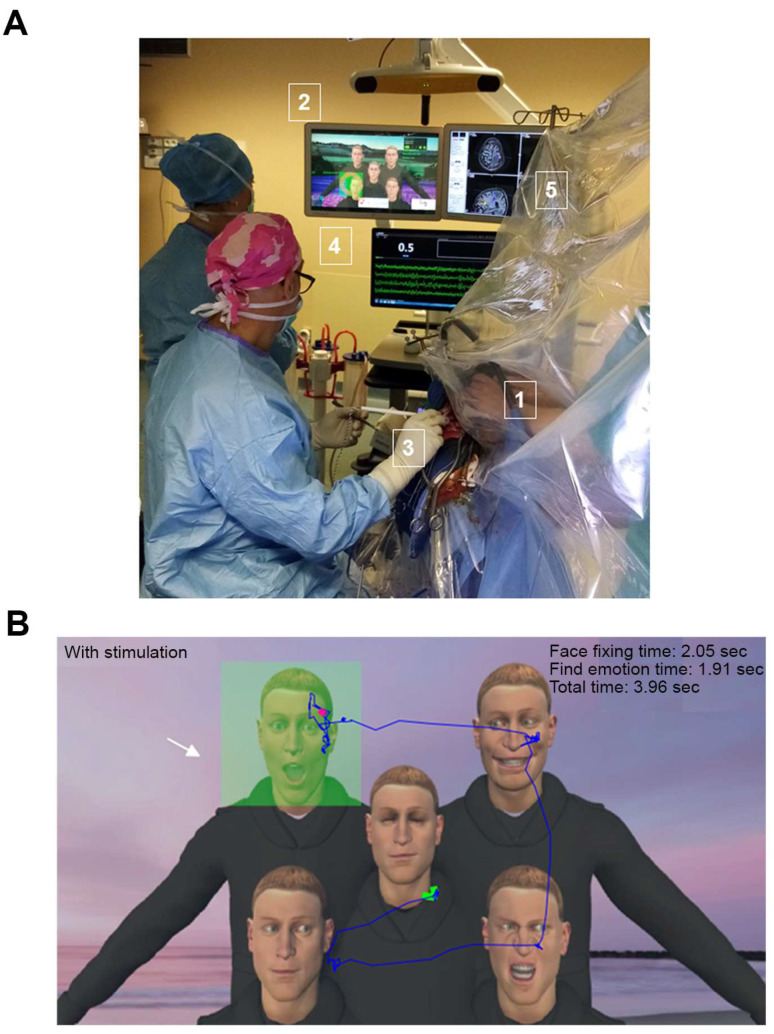
Intraoperative procedure with VR tasks. (**A**) View of the operative room during the procedure (1: head of the patient, wearing the VRH; 2: screen showing what the patient is seeing in the VRH, his gaze materialized by a green spot; 3: application of DES to the exposed brain; 4: screen showing EEG signals; 5: neuronavigation showing white matter fascicles and the position of the electrode). (**B**) Example of the VR task simulating a social interaction (VR-TANGO). The patient is asked to search for the avatar trying to make visual contact and to indicate the emotion expressed on the avatar’s face (joy, surprise or anger). The patient’s gaze is indicated by a blue line. The green square indicates the avatar making eye contact. The arrow indicates the avatar that the patient gazed at for more than 0.6 sec (thereby triggering the dynamic facial emotion). In this example, the patient identified the avatar making eye contact in 2.05 sec and indicated the emotion expressed 1.91 sec later. (EEG, electroencephalogram; VRH, virtual reality headset).

VR applications have progressed at a similar rate to VRHs. We initially used non-dedicated VR applications developed as entertainment [37,38,39,40] or to simulate social interactions, with the social platform vTime^®^ [35,41]. We then developed three types of dedicated applications. The first VR application was developed from the DO80 picture-naming test widely used to assess language function during AC (VR-DO80) [36,37]. The second VR application was developed from the Esterman test for intraoperative visual field assessment during AC (VR-Esterman) [13,15]. The Esterman test is the current gold standard for testing binocular visual fields and is used officially for driving license authorization in accordance with European recommendations. The third VR application developed was an interactive application called VR-TANGO (task for awake neurosurgery exploring gaze and TOM), which was developed for the simultaneous assessment of visuospatial exploration and detection of social cues, such as low-level TOM, including gaze processing and emotion recognition. The scene displayed by the VRH shows five avatars in front of a variable background (Figure 1A,B). One of the avatars is in the center and the others are located in the four quadrants of the VRH field visualized. Each avatar has a different eye-gaze direction. The patients are asked to identify the avatar making eye contact with them. The avatar expresses a dynamic facial emotion 0.6 sec after the patient establishes visual contact with the avatar to be found or stares at any other avatar for longer than 0.6 sec. The patient must identify the emotion expressed: joy, surprise or anger. The patient can also describe their assessment of the avatar’s intention to communicate (mental state attribution). The four quadrants of the visual field and all the emotions (joy, surprise or anger) were presented at random to patients.

### 2.4. Operative Procedure

The procedure used has been described in detail elsewhere [36,37]. Before surgery, all patients underwent a neuropsychological evaluation and fMRI, and were trained in the use of VR applications. The operating room organization is depicted in Figure 1A; it provides the neurosurgeon with real-time feedback for the VR task. The entire AC procedure was performed in the presence of an engineer, a neuropsychologist and/or speech therapist. An additional technical resource was needed to place and maintain the VRH on the patient’s head during VR tasks. Under general anesthesia, the patient was positioned in a supine or lateral position, according to the location of the tumor, with a rigid pin fixation of the head. Once the craniotomy, guided by neuro-navigation, was complete and the dura had been opened, the patient was awakened. Cortical DES was performed with a bipolar electrode delivering a biphasic current in association with motor and/or language tasks, such as the DO80 picture-naming task, performed using a computer tablet. Movement and/or spontaneous language were continuously monitored during tumor resection and a second mapping with subcortical DES was performed if necessary. VR applications were proposed during cortical or subcortical DES, and sometimes during the closure time, to distract the patient. The use of a typical task on a computer tablet or through VRH during AC is highly “personalized medicine” and the tasks were selected before surgery (during presurgical planning) with the neuropsychologist and the speech therapist. Heart rate, blood pressure and electroencephalogram (EEG) signals were recorded continuously during the procedure.

## 3. Results

In total, we evaluated VR tasks performed during AC for 64 patients: 37 men and 27 women, with a median age of 51 years (range: 23 to 75 years) (Table 1). Five of these patients underwent two operations: four for revision surgery and one for metastases in both cerebral hemispheres. Six patients were left-handed. The location of lesions was frontal (*n* = 31), parietal (*n* = 14), temporal (*n* = 3), occipital (*n* = 2), insular (*n* = 1), temporo-parietal (*n* = 8) and fronto-temporo-insular (*n* = 5). The lesions were of the following types: glioblastoma (*n* = 21), anaplastic astrocytoma (*n* = 16), anaplastic oligodendroglioma (*n* = 9), oligodendroglioma grade 2 (*n* = 3), metastasis (*n* = 14) and benign cystic (*n* = 1). AC was performed on the right hemisphere in 29 (42.0%) of the 69 operations performed. The median duration of surgery was 2 h 17 min (first incision to closing) and the median duration of the awake phase was 1 h 39 min. The median intensity of DES was 2 mA (range: 0.5 mA–8 mA). In total, one AC (1.5%) was performed with all three VR tasks (VR-DO80, VR-Esterman and VR-TANGO), 14 ACs (20.3%) were performed with two VR tasks and 54 ACs (78.3%) were performed with one VR task. The median duration of VRH use per patient was 15.5 min. The length of surgery with the use of VR tasks was similar to that previously reported with “traditional tests” (median (range): 2 h 43 min (1 h 28 min–5 h 25 min)) [42]. When we first began using this technique, we encountered difficulties positioning the VRH on the patient’s face due to the head holder. These difficulties were overcome by carefully positioning the VRH before the head holder, and before drawing the incision line. None of the patients experienced “VR sickness” during the 69 ACs. Intraoperative seizures of no consequence and unrelated to VRH were observed in 20.3% of cases. Some of the patients for whom VR was used as a relaxation tool during closure described it as pleasant and able to decrease pain and anxiety. Nevertheless, we have stopped using these applications during closure because the patients are generally exhausted at the end of the mapping phase and prefer to fall asleep.

**Figure 2:**
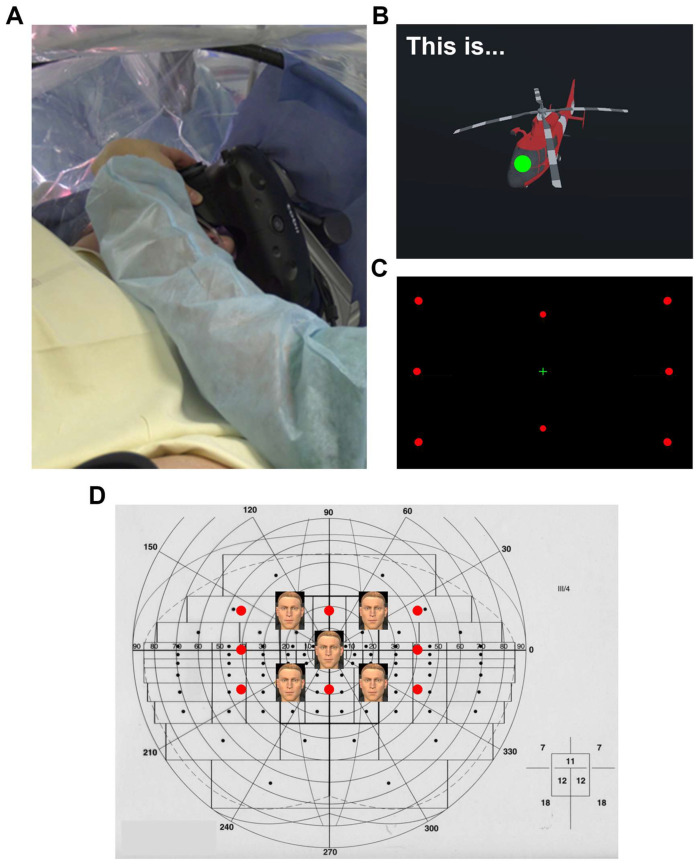
The VR-DO80 and VR-Esterman tests using the VRH combined with an eye-tracking device. (**A**) Patient wearing the VRH. (**B**) VR-DO80 naming task presented in 3D with the VRH. The green spot indicates the patient’s gaze. (**C**) VR-Esterman test exploring a visual field of 80° with 8 possible red dots on a black background around the green central visual axis. (**D**) The same dots projected on an Esterman grid on which the five avatars of the VR-TANGO are also positioned.

VR-DO80 was applied in 42 ACs (60.9%) (Table 1), and two versions were used with the VRH (Figure 2A). One version was two-dimensional and included the same images as the DO80 presented with a computer tablet (an image accompanied by the sentence “this is…”). The second version included the same items, but in stereoscopy, rotating in an empty virtual space (Figure 2B). Eye tracking data showed that patients never looked at the sentence “this is…”. This observation suggests that the patient “said” the sentence to themselves automatically, focusing only on the naming task. We observed that some areas for which the result was unclear during DES with the computer tablet (hesitation or delay in denomination) were clearly not eloquent areas during mapping with the VRH [36,37].

Visual field assessment was performed using the VR-Esterman task in 10 ACs (14.5%). This procedure was reserved for rare situations, such as lesions close to optical radiations, patients with a pre-existing scotome or blindness of the contralateral eye, and patients for whom a normal visual field was essential for their professional activity. The first VR-Esterman task used was performed using an Oculus VRH [13]. We then adapted this software to the HTC Vive combined with an eye-tracking device, to explore a visual field of 80° with eight possible red dots on a black background (Figure 2C,D). The maximum visual field attainable in the HTC Vive is 110°. However, we chose to retain the foam and not to press the VRH as close to the face as possible, so as to maximize user comfort. This configuration gave a visual field of 80°. Eye tracking made it possible to track the patient’s gaze accurately and to ensure that the patient really did focus on the central visual axis during visual field testing, without eye saccades. The VR-Esterman task made it possible to identify optical radiations. DES generated “positive” and “negative” phenomena. The positive phenomena were phosphenes in the corresponding contralateral visual field, which were easy for the patient to detect on the black background in the VRH. The negative phenomena were scotoma, with non-visualization of the red dot in the visual field tested.

**Figure 3:**
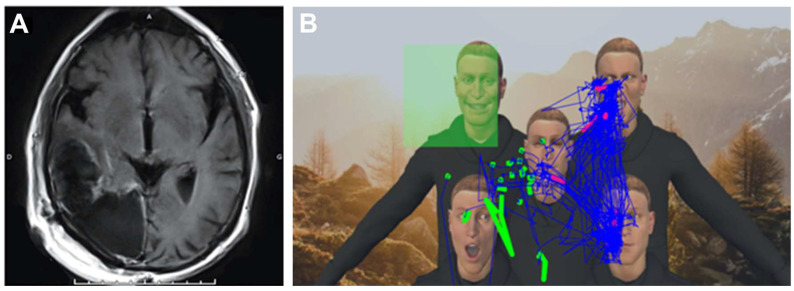
(**A**) Postoperative MRI scan for a patient with malignant right occipital glioma. The patient presented with homonymous hemianopia and USN. (**B**) Postoperative bedside VR-TANGO test: eye-tracking feedback for 10 cumulative tests. The patient was asked to determine the emotion expressed by the face looking at him (here, the upper left face, indicated by the green square). The eye tracking data recorded for the patient (blue lines) showed that he was unable to direct his attention to the left side. (USN, unilateral spatial neglect).

The VR-TANGO task was applied during 33 ACs (47.8%). By associating this task with the eye tracking data, we were able to detect difficulties exploring the space, difficulties locating the face of the avatar trying to make eye contact, and failures to recognize the facial emotion, resulting in a delay or a lack of response from the patient. During bedside evaluations, we found that the VR-TANGO task was not affected by homonymous hemianopia or other visual field defects, such as quadrantanopia or bitemporal hemianopia. Conversely, performance in this task was impaired in patients with USN and/or difficulties with emotion recognition or attention (Figure 3A,B).

**Figure 4:**
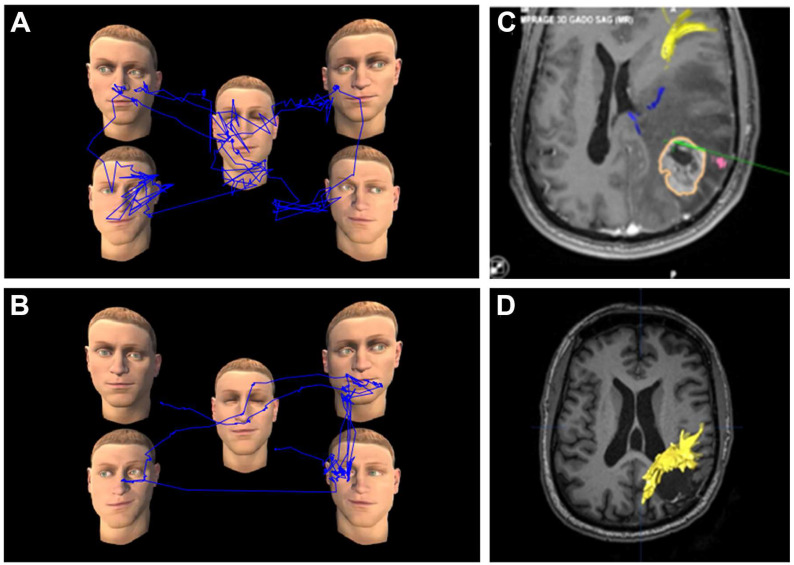
Resection of a malignant glioma located in the right temporo-parietal junction. (**A**) Intraoperative test without DES, with a normal performance. Gaze exploration, materialized by the blue line, indicated that the patient was able to explore the entire VRH screen and to find the avatar making eye contact (here, in the lower left corner). (**B**) Intraoperative test during DES of the white matter on the resection cavity wall, with an impaired performance. The patient had difficulties exploring the left part of the screen and was not able to find the avatar making eye contact (here, in the upper left corner). (**C**) Neuronavigation view showing the location of the DES electrode in contact with the SLF, not entirely visualized due to the peritumoral edema. (**D**) Postoperative tractography confirming the location of the electrode in contact with the SLF. (DES, direct electrical stimulation; SLF, superior longitudinal fasciculus).

**Figure 5:**
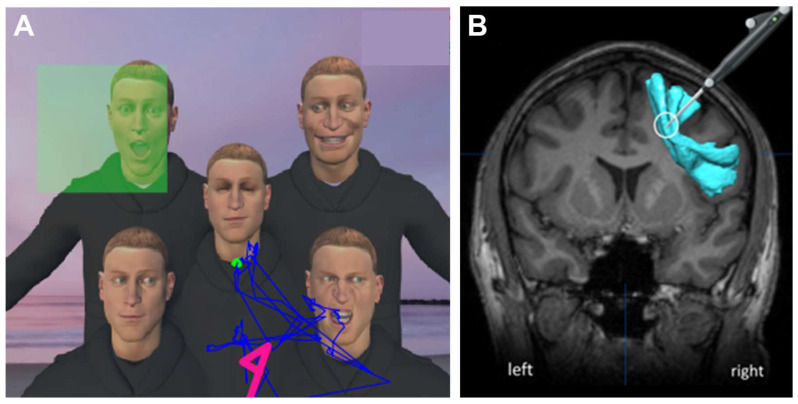
Resection of a malignant glioma in the frontal lobe. (**A**) VR-TANGO test during the DES. The patient was unable to detect the communicative cues, namely, eye contact (green square at the top left). His gaze remained fixed on the first face encountered. (**B**) Coronal neuronavigation showing the location of the DES electrode (white circle) in contact with the FAT (blue). (DES, direct electrical stimulation; FAT, frontal aslant tract).

During AC, several defects induced by subcortical DES with the VR-TANGO task were observed. For example, DES of the superior longitudinal fasciculus (SLF) induced mild USN, with the patient having difficulty exploring the left part of the VR screen (Figure 4B–D). Without DES, the patient was able to explore the entire VR screen in gaze exploration (Figure 4A). During DES of the inferior longitudinal fasciculus (ILF), one patient easily identified the correct avatar, but was unable to analyze or describe the facial emotion expressed (data not shown). This is not surprising, because the ILF and other ventral tracts have already been implicated in the recognition of facial emotions [43]. When the frontal aslant tract (FAT) was stimulated intraoperatively, one patient ignored the communicative cues. The gaze of this patient remained fixed on the first avatar encountered or went from face to face, with the patient unable to identify the avatar making eye contact or expressing a facial emotion (Figure 5A,B).

## 4. Discussion

This retrospective study shows that VR opens up new possibilities for the mapping of language, visual field and complex cognitive functions during AC. In previous studies, we demonstrated the feasibility and safety of the VR procedure [36,37]. The use of a VRH during AC does not specifically increase the rate of intraoperative seizures. Nevertheless, several precautions should be taken when using a VRH for brain mapping procedures, including the performance of these procedures by a well-trained team, with a well-trained patient and, despite the lack of consensus concerning its utility, intraoperative monitoring of brain electrical activity. The setup described here requires no additional hardware in the operating room other than the VRH, which is affordable for most neurosurgery centers. Its use adds to surgery time but, as for conventional awake testing (one set of the TANGO tasks takes five sec to perform), it does not significantly disrupt the surgical workflow.

We showed that the DO80 picture naming test and the Esterman test, the gold standard for language and visual field assessment, respectively, can be adapted to VRH combined with eye tracking. Patients were able to perform these VR tasks during surgery. Eye tracking was functional, making it possible to trace the gaze of the patient during the tasks. An exploration of 40° on either side of the central visual axis could be explored during the VR-Esterman task. This exploration does not meet all the French criteria for testing for driving licenses but can detect important visual defects that may impair quality of life. The technical features of VRHs will undoubtedly improve in the future, and it will probably soon be possible to explore a wider visual field with these devices. The adaptation of other language tasks, such as text-reading language tasks, for VRHs combined with eye tracking would be potentially interesting. Furthermore, we recently showed that, even several years after the end of treatment, adult survivors of primary brain tumor may experience daily life behavioral disorders induced by executive impairments with a negative effect on their health-related quality of life [44]. This highlights the potential benefits of adapting VRH combined with eye tracking for tasks exploring executive functions, such as the Stroop test [45].

The DO80 picture naming test and the Esterman test were easy to adapt for the VRH combined with eye tracking, but we encountered more difficulties for the adaptation of neuropsychological tasks evaluating visuospatial and social cognition. Various tests are used to assess these functions during AC. The line bisection test for visuospatial neglect [46] is widely used, because it is simple, rapid and reproducible [20,26,47,48,49,50]. However, even though this task identifies a profound perceptual disorder, it appears to be anatomically and behaviorally independent of the core symptoms of neglect. In 40% of patients with core symptoms of spatial neglect, no impairment is observed in the line bisection task. One possible explanation for this dissociation is that the line bisection task draws on allocentric representation, whereas the core deficit in spatial neglect is egocentric [51,52,53]. Another allocentric test, the target cancelation task, which involves searching for and crossing out target symbols, is more sensitive than the line bisection task for USN detection, but less widely used during AC [16]. For evaluations of social cognition, facial emotion recognition tasks are frequently performed during AC, based on photographs of individuals displaying one of the six primary facial emotions (anger, happiness, fear, surprise, disgust and sadness) extracted from several tests (Ekman’s Faces, the Brief Affect Recognition Test, the Japanese and Caucasian Brief Affective Recognition Test, and the ATR facial expression database) [18,54,55]. Published results suggest that, when presented for 10 sec at the patient’s bedside, the emotion portrayed by each photograph is correctly identified by more than 70% of patients. In our experience, tests involving the recognition of facial emotions from photographs are difficult to perform during AC, with a high rate of error, even in the absence of DES. Dynamic facial expressions are more accurately recognized than static expressions [56,57]. A simplified and adapted “reading the mind in the eyes test” (RMET) based on photographs centered on the eye region is also performed in patients undergoing AC for right-side low-grade gliomas, to explore TOM [28,58]. However, RMET appears to measure emotion recognition rather than TOM ability [59].

Rather than immediately developing a specific application to test and map social cognition, we initially evaluated the potential of the available VR social networks, such as the social VR application vTime^®^ [35]. This application simulates virtual social interactions with an avatar controlled by the neuropsychologist, who also wears a VRH. However, we experienced several limitations of the use of the social VR application vTime^®^, precisely because of the lack of control of all potent non-verbal language cues, including facial expressions and eye gaze. We therefore decided to pursue our efforts to explore visuospatial cognition and non-verbal language during AC by developing an interactive VR application capable of analyzing these functions simultaneously. This project was not an easy one, but the solution was found in Argentinian tango dancing (a hobby of one of the authors). The invitation to dance the tango is highly codified, lasting only a few sec and including spatial exploration of the dance hall, tracking someone who looks at you, social attention fixed on a partner but ready to be reoriented if gazed at by someone else, analysis of faces, interpretations of the emotions of the other person and guessing what he/she thinks from his/her movements, his/her desire to be invited to dance and his/her probable reaction. We adapted this short scenario with VR technology and named this task “TANGO”.

The development of a program of this type was challenging, for several reasons, including the need for the animation to appear natural. A neutral face, capable of producing a smile at the same time as a slight movement of the head, associated with a gaze capable of making eye contact with the patient, must be achieved with professional motion capture tools. We achieved this in collaboration with a group of engineers using techniques from video games and the movie industry, in which facial animation is achieved by filming an actor and transposing his or her movements onto the avatars (“Hardware—Dynamixyz”). We aimed to reproduce a social scene by having several avatars in a virtual world. However, although faces have a spatial advantage for capturing attention, reflecting their particular saliency and their social value, the maximum number of faces that can be analyzed in a visual field of 110° in less than five sec (the maximum time for DES) is five. The scene was therefore designed to include four avatars in the four corners of the visualized field. The patient was asked to search for the avatar trying to make visual contact and to describe the automatically triggered facial emotion of the avatar or their feelings about the desire for communication/social contact as expressed by the avatar. The preliminary results with this application showed that, unlike the line bisection task, the VR-TANGO test was unaffected by homonymous hemianopia [60]. By contrast, the performance of the TANGO task was impaired by USN, with the patient unable to direct attention to the left side. The TANGO task can be seen as a cancelation test with distractors, and is therefore very sensitive for the detection of USN, probably more so than the line bisection test. We observed that the DES of some subcortical areas of the brain affected the response to this test. For example, when the right FAT was transiently disrupted by DES, the patient’s gaze shifted from one face to another, unable to identify which avatar was trying to make eye contact, or remained fixed on the first avatar encountered regardless of emotion or intent to communicate. The involvement of the FAT in the expression and recognition of communicative intentions may account for this behavior [61,62,63]. The right FAT has a putative role in the support of executive function, through inhibitory control and conflict monitoring for action, which may provide an alternative explanation [64,65]. These preliminary data indicate that VR-TANGO may be useful for identifying the various components of prelinguistic social abilities and their neural substrates.

## 5. Limitations

The main limitation of this study is that it does not provide clinical results for VR tasks during AC in terms of test performance, clinical preservation and extent of resection. Several established bedside tests are currently being performed to evaluate the performance of VR tasks before their use in clinical practice for brain tumor resection in awake patients (ClinicalTrials.gov NCT04288505). For example, the bells test [66], the Ekman test [67] and the RMET [68] are being used to assess the performance of the VR-TANGO task for detecting visuospatial and social cognition impairments. Once this validation is complete, it will be possible to perform prospective studies to assess the benefits of using VR tasks during AC to preserve language, visual field, visuospatial and social cognition or, more globally, the ability to perform daily life activities.

## 6. Conclusions

VR has a unique advantage over other techniques, in that it provides neurosurgeons with a way of investigating various complex functions, including non-verbal cognition, that would not otherwise be possible in the operating room. The use of virtual environments advocated here should not minimize the contribution of traditional pen-and-paper tasks and/or computer screens/tablets, which have advantages for brain mapping. Eye trackers in VRHs, combining the features of both mobile and remote setups, minimize the risk of calibration loss and improve success rates for the measurement of eye positions and movements. Furthermore, the use of a VRH immerses the patient in the VR task through complete isolation from the surrounding operating room. We acknowledge that the VR-TANGO task we have developed is not deeply immersive. We are currently working on stereoscopic sound and smells, to make the virtual experience more immersive and emotionally engaging. The patient could be immersed in the environment of a coffee bar, for example with the aroma of hot coffee, street sounds and people sitting at neighboring tables. It will soon be possible to use “deep fake” technology to replace the faces of avatars with those of the patient’s friends or family. It will also be possible, in the near future, to map social cognition without the need for verbal participation from the patient. The new VRH technologies are making it possible to track the participant’s behavior and reactions to the VR experience by synchronizing various sources of data, to obtain a holistic, integrated measurement of target social interactions through machine-learning techniques. The most studied data after eye tracking are facial mimicry, pupillometry and electroencephalographic coherence data. Given the speed of the progress in VR technology and the unbelievable sense of immersion given by the latest devices, ethical questions are beginning to arise. Continuous reflection and discussions of ethical and methodological considerations are required concerning the use of these advanced technologies in brain mapping procedures during AC.

## Figures and Tables

**Table 1:** Characteristics of the 64 patients and the 69 AC procedures (AC, awake craniotomy).

Patients (*n* = 64)		*n*	%
**Age (years)**	median (range): 51 years (23 years–75 years)		
<70		61	95.3
≥70		3	4.7
**Sex**			
Male		37	57.8
Female		27	42.2
**Handedness**			
Right		58	90.6
Left		6	9.4
**Tumor location**			
Hemisphere			
Left		38	59.4
Right		26	40.6
Lobe			
Frontal		31	48.4
Temporal		3	4.7
Parietal		14	21.9
Occipital		2	3.1
Insular		1	1.6
Temporo-parietal junction		8	12.5
Fronto-temporo-insular		5	7.8
**Tumor histology**			
Glioblastoma		21	32.8
Anaplastic astrocytoma		16	25.0
Anaplastic oligodendroglioma		9	14.1
Oligodendroglioma grade 2		3	4.7
Metastasis		14	21.9
Benign cystic lesion		1	1.6
**Awake surgery (*n* = 69)**		***n***	**%**
**Duration of surgery**	median (range): 2 h 17 min (1 h–5 h 20 min)		
**Time awake**	median (range): 1 h 39 min (38 min–4 h 30 min)		
**Intensity of stimulation (mA)**	median (range): 2.0 mA (0.5 mA–8.0 mA)		
**Intraoperative seizures**		14	20.3
**Hemisphere location**			
Left		40	58.0
Right		29	42.0
**Duration of VRH use per patient**	median (range): 15.5 min (3.0 min–53.0 min)		
**VR tasks**			
Number per AC			
One task		54	78.3
Two tasks		14	20.3
Three tasks		1	1.5
Type			
VR-DO80		42	60.9
VR-TANGO		33	47.8
VR-Esterman		10	14.5

## Data Availability

The datasets generated and/or analyzed in this study are available from the corresponding author under the authorization of the delegation for clinical research and innovation (DRCI, CHU, Angers).

## Abbreviations

DES, direct electrical stimulation; SLF, superior longitudinal fasciculus; TANGO, task for awake neurosurgery exploring gaze and TOM; TOM, theory of mind; UN, unilateral neglect; VR, virtual reality; VRH, virtual reality headset.

## References

[1] Duffau H. (2021). New Philosophy, Clinical Pearls, and Methods for Intraoperative Cognition Mapping and Monitoring “à La Carte” in Brain Tumor Patients. Neurosurgery.

[2] Martín-Monzón I., Rivero Ballagas Y., Arias-Sánchez S. (2022). Language Mapping: A Systematic Review of Protocols That Evaluate Linguistic Functions in Awake Surgery. Appl. Neuropsychol. Adult.

[3] Morshed R.A., Young J.S., Lee A.T., Berger M.S., Hervey-Jumper S.L. (2021). Clinical Pearls and Methods for Intraoperative Awake Language Mapping. Neurosurgery.

[4] Motomura K., Ohka F., Aoki K., Saito R. (2022). Supratotal Resection of Gliomas With Awake Brain Mapping: Maximal Tumor Resection Preserving Motor, Language, and Neurocognitive Functions. Front. Neurol..

[5] Seidel K., Szelényi A., Bello L. (2022). Intraoperative Mapping and Monitoring during Brain Tumor Surgeries. Handb. Clin. Neurol..

[6] Mandonnet E., Cerliani L., Siuda-Krzywicka K., Poisson I., Zhi N., Volle E., de Schotten M.T. (2017). A Network-Level Approach of Cognitive Flexibility Impairment after Surgery of a Right Temporo-Parietal Glioma. Neurochirurgie.

[7] Sagberg L.M., Iversen D.H., Fyllingen E.H., Jakola A.S., Reinertsen I., Solheim O. (2019). Brain Atlas for Assessing the Impact of Tumor Location on Perioperative Quality of Life in Patients with High-Grade Glioma: A Prospective Population-Based Cohort Study. NeuroImage Clin..

[8] Salo J., Niemelä A., Joukamaa M., Koivukangas J. (2002). Effect of Brain Tumour Laterality on Patients’ Perceived Quality of Life. J. Neurol. Neurosurg. Psychiatry.

[9] Weed E., McGregor W., Feldbaek Nielsen J., Roepstorff A., Frith U. (2010). Theory of Mind in Adults with Right Hemisphere Damage: What’s the Story?. Brain Lang..

[10] Bernard F., Lemée J.-M., Ter Minassian A., Menei P. (2018). Right Hemisphere Cognitive Functions: From Clinical and Anatomic Bases to Brain Mapping During Awake Craniotomy Part I: Clinical and Functional Anatomy. World Neurosurg..

[11] Fortin D., Iorio-Morin C., Tellier A., Goffaux P., Descoteaux M., Whittingstall K. (2021). High-Grade Gliomas Located in the Right Hemisphere Are Associated With Worse Quality of Life. World Neurosurg..

[12] Wang H., Zhao P., Zhao J., Zhong J., Pan P., Wang G., Yi Z. (2022). Theory of Mind and Empathy in Adults With Epilepsy: A Meta-Analysis. Front. Psychiatry.

[13] Mazerand E., Le Renard M., Hue S., Lemée J.-M., Klinger E., Menei P. (2017). Intraoperative Subcortical Electrical Mapping of the Optic Tract in Awake Surgery Using a Virtual Reality Headset. World Neurosurg..

[14] Santos C., García V., Gómez E., Velásquez C., Martino J. (2022). Visual Mapping for Tumor Resection: A Proof of Concept of a New Intraoperative Task and A Systematic Review of the Literature. World Neurosurg..

[15] Menei P., Clavreul A., Casanova M., Colle D., Colle H., Mandonnet E., Herbet G. (2021). Vision. Intraoperative Mapping of Cognitive Networks.

[16] Conner A.K., Glenn C., Burks J.D., McCoy T., Bonney P.A., Chema A.A., Case J.L., Brunner S., Baker C., Sughrue M. (2016). The Use of the Target Cancellation Task to Identify Eloquent Visuospatial Regions in Awake Craniotomies: Technical Note. Cureus.

[17] Fried I., Mateer C., Ojemann G., Wohns R., Fedio P. (1982). Organization of Visuospatial Functions in Human Cortex. Evidence from Electrical Stimulation. Brain J. Neurol..

[18] Giussani C., Pirillo D., Roux F.-E. (2010). Mirror of the Soul: A Cortical Stimulation Study on Recognition of Facial Emotions. J. Neurosurg..

[19] Herbet G., Moritz-Gasser S. (2019). Beyond Language: Mapping Cognition and Emotion. Neurosurg. Clin. N. Am..

[20] Kitabayashi T., Nakada M., Kinoshita M., Sakurai H., Kobayashi S., Okita H., Nanbu Y., Hayashi Y., Hamada J.-I. (2012). Awake surgery with line bisection task for two cases of parietal glioma in the non-dominant hemisphere. No Shinkei Geka.

[21] Lemée J.-M., Bernard F., Ter Minassian A., Menei P. (2018). Right Hemisphere Cognitive Functions: From Clinical and Anatomical Bases to Brain Mapping During Awake Craniotomy. Part II: Neuropsychological Tasks and Brain Mapping. World Neurosurg..

[22] Nakada M., Nakajima R., Okita H., Nakade Y., Yuno T., Tanaka S., Kinoshita M. (2021). Awake Surgery for Right Frontal Lobe Glioma Can Preserve Visuospatial Cognition and Spatial Working Memory. J. Neurooncol..

[23] Nakajima R., Kinoshita M., Okita H., Liu Z., Nakada M. (2021). Preserving Right Pre-Motor and Posterior Prefrontal Cortices Contribute to Maintaining Overall Basic Emotion. Front. Hum. Neurosci..

[24] Prat-Acín R., Galeano-Senabre I., López-Ruiz P., Ayuso-Sacido A., Espert-Tortajada R. (2021). Intraoperative Brain Mapping of Language, Cognitive Functions, and Social Cognition in Awake Surgery of Low-Grade Gliomas Located in the Right Non-Dominant Hemisphere. Clin. Neurol. Neurosurg..

[25] Puglisi G., Sciortino T., Rossi M., Leonetti A., Fornia L., Conti Nibali M., Casarotti A., Pessina F., Riva M., Cerri G. (2018). Preserving Executive Functions in Nondominant Frontal Lobe Glioma Surgery: An Intraoperative Tool. J. Neurosurg..

[26] Roux A., Lemaitre A.-L., Deverdun J., Ng S., Duffau H., Herbet G. (2021). Combining Electrostimulation With Fiber Tracking to Stratify the Inferior Fronto-Occipital Fasciculus. Front. Neurosci..

[27] Rutten G.-J.M., Landers M.J.F., De Baene W., Meijerink T., van der Hek S., Verheul J.H.B. (2021). Executive Functional Deficits during Electrical Stimulation of the Right Frontal Aslant Tract. Brain Imaging Behav..

[28] Yordanova Y.N., Cochereau J., Duffau H., Herbet G. (2019). Combining Resting State Functional MRI with Intraoperative Cortical Stimulation to Map the Mentalizing Network. NeuroImage.

[29] Rahimpour S., Haglund M.M., Friedman A.H., Duffau H. (2019). History of Awake Mapping and Speech and Language Localization: From Modules to Networks. Neurosurg. Focus.

[30] Pallud J., Rigaux-Viode O., Corns R., Muto J., Lopez Lopez C., Mellerio C., Sauvageon X., Dezamis E. (2017). Direct Electrical Bipolar Electrostimulation for Functional Cortical and Subcortical Cerebral Mapping in Awake Craniotomy. Practical Considerations. Neurochirurgie.

[31] Katsevman G.A., Greenleaf W., García-García R., Perea M.V., Ladera V., Sherman J.H., Rodríguez G. (2021). Virtual Reality During Brain Mapping for Awake-Patient Brain Tumor Surgery: Proposed Tasks and Domains to Test. World Neurosurg..

[32] Mishra R., Narayanan M.D.K., Umana G.E., Montemurro N., Chaurasia B., Deora H. (2022). Virtual Reality in Neurosurgery: Beyond Neurosurgical Planning. Int. J. Environ. Res. Public. Health.

[33] Parsons T.D., Gaggioli A., Riva G. (2017). Virtual Reality for Research in Social Neuroscience. Brain Sci..

[34] Vayssiere P., Constanthin P.E., Herbelin B., Blanke O., Schaller K., Bijlenga P. (2022). Application of Virtual Reality in Neurosurgery: Patient Missing. A Systematic Review. J. Clin. Neurosci. Off. J. Neurosurg. Soc. Australas..

[35] Bernard F., Lemée J.-M., Aubin G., Ter Minassian A., Menei P. (2018). Using a Virtual Reality Social Network During Awake Craniotomy to Map Social Cognition: Prospective Trial. J. Med. Internet Res..

[36] Casanova M., Clavreul A., Soulard G., Delion M., Aubin G., Ter Minassian A., Seguier R., Menei P. (2021). Immersive Virtual Reality and Ocular Tracking for Brain Mapping During Awake Surgery: Prospective Evaluation Study. J. Med. Internet Res..

[37] Delion M., Klinger E., Bernard F., Aubin G., Minassian A.T., Menei P. (2020). Immersing Patients in a Virtual Reality Environment for Brain Mapping during Awake Surgery: Safety Study. World Neurosurg..

[38] Ocean Rift sur Oculus Rift [Internet] Oculus. https://www.oculus.com/experiences/rift/1253785157981619/.

[39] VR Projects [Internet] Julius Horsthuis. http://www.julius-horsthuis.com/vr-projects.

[40] Zen Parade—Shape Space VR [Internet]. http://www.shapespacevr.com/zen-parade.html.

[41] vTime: The VR Sociable Network—Out Now for Windows Mixed Reality, Gear VR, Oculus Rift, iPhone, Google Daydream, and Google Cardboard [Internet]. http://www.webcitation.org/6zKYC8j6Q.

[42] Clavreul A., Aubin G., Delion M., Lemée J.-M., Ter Minassian A., Menei P. (2021). What Effects Does Awake Craniotomy Have on Functional and Survival Outcomes for Glioblastoma Patients?. J. Neurooncol..

[43] Herbet G., Zemmoura I., Duffau H. (2018). Functional Anatomy of the Inferior Longitudinal Fasciculus: From Historical Reports to Current Hypotheses. Front. Neuroanat..

[44] Cantisano N., Menei P., Roualdes V., Seizeur R., Allain P., Le Gall D., Roy A., Dinomais M., Laurent A., Besnard J. (2021). Relationships between Executive Functioning and Health-Related Quality of Life in Adult Survivors of Brain Tumor and Matched Healthy Controls. J. Clin. Exp. Neuropsychol..

[45] Stroop J.R. (1935). Studies of Interference in Serial Verbal Reactions. APA PsycArticles.

[46] Schenkenberg T., Bradford D.C., Ajax E.T. (1980). Line Bisection and Unilateral Visual Neglect in Patients with Neurologic Impairment. Neurology.

[47] Talacchi A., Squintani G.M., Emanuele B., Tramontano V., Santini B., Savazzi S. (2013). Intraoperative Cortical Mapping of Visuospatial Functions in Parietal Low-Grade Tumors: Changing Perspectives of Neurophysiological Mapping. Neurosurg. Focus.

[48] Vallar G., Bello L., Bricolo E., Castellano A., Casarotti A., Falini A., Riva M., Fava E., Papagno C. (2014). Cerebral Correlates of Visuospatial Neglect: A Direct Cerebral Stimulation Study. Hum. Brain Mapp..

[49] Rolland A., Herbet G., Duffau H. (2018). Awake Surgery for Gliomas within the Right Inferior Parietal Lobule: New Insights into the Functional Connectivity Gained from Stimulation Mapping and Surgical Implications. World Neurosurg..

[50] Bartolomeo P., Thiebaut de Schotten M., Duffau H. (2007). Mapping of Visuospatial Functions during Brain Surgery: A New Tool to Prevent Unilateral Spatial Neglect. Neurosurgery.

[51] Karnath H.O., Ferber S., Himmelbach M. (2001). Spatial Awareness Is a Function of the Temporal Not the Posterior Parietal Lobe. Nature.

[52] Rorden C., Fruhmann Berger M., Karnath H.-O. (2006). Disturbed Line Bisection Is Associated with Posterior Brain Lesions. Brain Res..

[53] Chechlacz M., Rotshtein P., Humphreys G.W. (2012). Neuroanatomical Dissections of Unilateral Visual Neglect Symptoms: ALE Meta-Analysis of Lesion-Symptom Mapping. Front. Hum. Neurosci..

[54] Papagno C., Pisoni A., Mattavelli G., Casarotti A., Comi A., Fumagalli F., Vernice M., Fava E., Riva M., Bello L. (2016). Specific Disgust Processing in the Left Insula: New Evidence from Direct Electrical Stimulation. Neuropsychologia.

[55] Motomura K., Terasawa Y., Natsume A., Iijima K., Chalise L., Sugiura J., Yamamoto H., Koyama K., Wakabayashi T., Umeda S. (2019). Anterior Insular Cortex Stimulation and Its Effects on Emotion Recognition. Brain Struct. Funct..

[56] Joyal C.C., Jacob L., Cigna M.-H., Guay J.-P., Renaud P. (2014). Virtual Faces Expressing Emotions: An Initial Concomitant and Construct Validity Study. Front. Hum. Neurosci..

[57] Krumhuber E.G., Kappas A., Manstead A.S.R. (2013). Effects of Dynamic Aspects of Facial Expressions: A Review. Emot. Rev..

[58] Yordanova Y.N., Duffau H., Herbet G. (2017). Neural Pathways Subserving Face-Based Mentalizing. Brain Struct. Funct..

[59] Oakley B.F.M., Brewer R., Bird G., Catmur C. (2016). Theory of Mind Is Not Theory of Emotion: A Cautionary Note on the Reading the Mind in the Eyes Test. J. Abnorm. Psychol..

[60] Kerkhoff G., Schenk T. (2011). Line Bisection in Homonymous Visual Field Defects—Recent Findings and Future Directions. Cortex J. Devoted Study Nerv. Syst. Behav..

[61] Catani M., Bambini V. (2014). A Model for Social Communication And Language Evolution and Development (SCALED). Curr. Opin. Neurobiol..

[62] Burkhardt E., Kinoshita M., Herbet G. (2021). Functional Anatomy of the Frontal Aslant Tract and Surgical Perspectives. J. Neurosurg. Sci..

[63] Gallet C., Clavreul A., Bernard F., Menei P., Lemée J.-M. (2022). Frontal Aslant Tract in the Non-Dominant Hemisphere: A Systematic Review of Anatomy, Functions, and Surgical Applications. Front. Neuroanat..

[64] Dick A.S., Garic D., Graziano P., Tremblay P. (2019). The Frontal Aslant Tract (FAT) and Its Role in Speech, Language and Executive Function. Cortex J. Devoted Study Nerv. Syst. Behav..

[65] Garic D., Broce I., Graziano P., Mattfeld A., Dick A.S. (2019). Laterality of the Frontal Aslant Tract (FAT) Explains Externalizing Behaviors through Its Association with Executive Function. Dev. Sci..

[66] Ferber S., Karnath H.O. (2001). How to Assess Spatial Neglect--Line Bisection or Cancellation Tasks?. J. Clin. Exp. Neuropsychol..

[67] Ekman P., Friesen W.V. (1976). Measuring Facial Movement. Environ. Psychol. Nonverbal Behav..

[68] Baron-Cohen S., Wheelwright S., Hill J., Raste Y., Plumb I. (2001). The “Reading the Mind in the Eyes” Test Revised Version: A Study with Normal Adults, and Adults with Asperger Syndrome or High-Functioning Autism. J. Child Psychol. Psychiatry.

